# Destination Imagery Diagnosis Model: The Case of Switzerland

**DOI:** 10.1007/978-3-030-65785-7_36

**Published:** 2020-11-28

**Authors:** Meng-Mei Chen, Laura Zizka, Effie Ruiheng Zhang, Justine Gentinetta

**Affiliations:** 1 Department for Informatics, Technical University of Munich, Garching bei München, Bayern Germany; 2 Smart Tourism Education Platform (STEP) College of Hotel and Tourism Management, Kyung Hee University, Seoul, Korea (Republic of); 3 Department of Tourism and Service Management, MODUL University Vienna, Vienna, Wien Austria; Ecole hoteliere de Lausanne, HES-SO University of Applied Sciences and Arts Western, Lausanne, Switzerland

**Keywords:** Destination Imagery Diagnosis model, Association strength, Association valence

## Abstract

This research investigates destination imagery of Switzerland as a travel destination. This research first conducted survey and content analysis to identify 23 unique statements reflecting travel in Switzerland. Through an online survey, this research collected 399 responses from French and Italian respondents. Based on the comparisons of association strength and association valence of every statement to the aggregated association strength and association valence, this research developed the Destination Imagery Diagnosis model. The results show that, overall, French and Italian respondents have strong and positive associations to statements related to Switzerland’s nature and opportunities for outdoor activities. Furthermore, respondents rated “Healthy lifestyle” and “Welcoming and friendly” positively but the associations to Switzerland were weaker. This research also identified marketing opportunities specifically for French and Italian respondents. The Destination Imagery Diagnosis Model serves as a new tool to compare destination imageries between markets or keep track of changes of destination imagery.

## Introduction

Previous researchers have documented that both cognitive and affective perceptions contribute to overall destination image, and destination image impacts on the intention to visit. On the other hand, although destination image is one of the most researched topics in tourism, many researchers challenged the ambiguity of the destination image construct [6, 9]. Researchers have advocated the delimitation of concepts between destination imagery and destination image [6], and defined destination imagery as “an individual’s or a group of individuals’ diverse cognitive and affective associations relating to a destination”; while destination image is “an overall evaluative representation of a destination”. Researchers have further proposed the Destination Content Model, which differs from the existing destination image model in replacing cognitive image and affective image with destination imagery, proposing destination affect as a new construct, as well as in measuring destination imagery, destination affect and destination image [8].

The objectives of this research are to understand the destination imagery of Switzerland, and to identify marketing opportunities. This research makes contributions to the academia by testing the destination imagery concept proposed in [6, 8], developing marketing insights based on the results, and further proposing a Destination Imagery Diagnosis model. This research also contributes to the practitioners by understanding destination imagery associated with Switzerland in target markets, and the findings could be used to evaluate and improve marketing and branding efforts to develop demands for Switzerland tourism.

## Literature Review

### Destination Image

Destination image is considered as an attitudinal construct consisting of an individual’s mental representation of knowledge, beliefs, feelings, and global impression about an object or destination [3, 5, 13]. Researchers have advocated that the measurement of destination image should consist of three dimensions, including functional and psychological characteristics, attributes and holistic (imagery), and common and unique attributes. Nevertheless, most destination image research focused on functional and psychological, and attributes and holistic dimensions [5].

Destination image construct contains two evaluations: perceptual/cognitive evaluation (the beliefs or knowledge about a destination’s attributes), and affective evaluation (feelings toward, or attachment to the destination) [3]. Generally, researchers believe the cognitive components impact on the affective components, hence these two constructs are different but hierarchically related [3]. Furthermore, cognitive image influences the overall image either directly or indirectly through affective image [3].

Commonly the measurement of cognitive image is to firstly identify attributes related to the studied destination through literature review, focus group interviews, and content analysis, then ask respondents to evaluate these attributes on semantic or Likert scales [3, 5, 13]. On the other hand, the measurement of affective components is through four bipolar items, including arousing-sleepy, pleasant-unpleasant, exciting-gloomy, and relaxing-distressing. Although these four bipolar items were used in numerous research, and researchers almost treated all other attributes as cognitive image, some researchers also raised questions. For example, researchers advocated destination image should include both functional and psychological characteristics, and gave the example of “friendliness” as the only psychological attribute measured by the majority of researchers [5]. Researchers have stated “…personal safety, friendliness, …can be considered as cognitive image attributes of a more psychological nature”, but still measured affective components with the popular four bipolar attributes [7, 8]. On the other hand, researchers have used text mining and sentiment analysis to analyse reviews in order to understand tourists’ impressions and feelings [1]. Yet, researchers also argued that destination affect should be an overall affective response or an overall affective state of like or dislike to a destination [6, 8].

### Destination Imagery

Although destination image is one of the most popular research topics, it has been criticized by different scholars for its ambiguity in the construct itself [4–6, 8, 9, 13]. An extensive review of the methodological issues related to destination image, including dimensionality (single vs. Multidimensional), and the nature of construct (formative or reflective, descriptive or evaluative) can be found in [6]. Consequently, researchers have proposed to differentiate destination image and destination imagery [6, 8]. Destination imagery is defined as “an individual’s or a group of individuals’ diverse cognitive and affective associations relating to a destination” [6, 8]. Destination imagery is descriptive in nature, and will benefit from using a formative modelling approach [6, 8].

On the other hand, destination image is defined as “a shortcut or outcome of the array of associations which people mentally link it to a destination”; or a mental shortcut that individuals use to make judgements and decision efficiently; and is evaluative in nature [6, 8].

### Research Questions

The differentiation between destination imagery and destination image clarifies some methodological issues such as the ambiguity of the definition and the nature of variables (formative vs. Reflective, descriptive vs. Evaluative). Furthermore, destination imagery aligns with the general psychology concepts including the Theory of Spreading Activation and memes [2], as well as work done by some tourism researchers in destination branding [4, 8]. Common memes could be used to activate potential customers associations, while unique memes could serve as competitive advantages [2]. Hence, memes associated with a destination, or destination imagery is critical to tourism marketing.

This research aims to understand the destination imagery of Switzerland in the target markets. As an exploratory research, this research will first identify the attributes associated with Switzerland, and then investigate the strength and valence of these associations perceived in the target markets.

## Method

### First Phase

Both content analysis and surveys have been conducted to identify the destination imagery associated with Switzerland tourism. The survey has three questions asking respondents to provide keywords associated with stereotypes, feelings, and uniqueness of Switzerland tourism [4, 5, 10], as well as demographic information. The survey was distributed on social media (LinkedIn), and on campus of a Swiss university between Spring and Fall 2019. In addition, articles related to Switzerland tourism from popular guidebook websites (e.g. Lonely Planet) and newspaper websites (BBC, New York Times) were downloaded.

Data from the online survey, mass media, and travel guidebook website were merged into a master file. Four researchers (one Swiss and three long-term Swiss residents) conducted content analysis independently to identify unique attributes. Similar to [8], the content analysis did not aim to generate frequency counts of popular words, but identify unique statements about Switzerland as a travel destination. The content analysis from four researchers were compared and finally a total of 23 statements were identified to be used in the second survey.

### Second Phase

For each statement, both association strength and association valence were measured. Association strength is measured with the question “how strongly do you associate this attribute to Switzerland?” while association valence is measured with the question “for you as a tourist in Switzerland, is this attribute positive or negative?” [8]. The association strength was measured in 5 points (1 not at all; 5 totally) while the association valence was measured with 7-point (−3 extremely negative; 3 extremely positive) Likert scales. The final survey consists of demographic questions, travel experience questions, and 23 attribute related questions. A total of 15 Swiss residents were invited to conduct the pilot test, and minor adjustments were made after the pilot test. Finally, the survey was translated and back-translated into French and Italy by native speakers.

This research used Prolific online panel to collect data. The main tourist original countries for Switzerland are Germany, the UK, France, Italy, and the US. Accordingly, this research aims to collect data from panellists with their nationality or country of residences in France or Italy. To improve the quality control, the approval rate of the panellist needs to be at least 90%. In addition, attention check questions were included in the survey.

## Results and Discussions

### Respondent Profile

The data collection took place in January 2020. After deleting respondents failed the attention tests, the numbers of respondents for France and Italy are 200 and 199, respectively. Table 1 presents the respondent profiles.

**Table 1.** Respondents profiles.

Description	France		Italy	
Age	Number	Percentage	Number	Percentage
18–29	133	67	140	69
30–39	38	19	42	21
40–49	17	9	11	6
50–59	9	5	5	3
60 and above	3	2	1	1
Total	200	100	199	100
Gender				
Male	101	51	124	62
Female	99	49	75	38
Total	200	100	199	100
Travel experience				
Never visited	108	54	125	63
Visited	92	46	71	27
Total	200	100	199	100

### Association Strength and Association Valence

The means of the association strength and association valence were presented in Table 2. The overall association strengths are 3.55 for French, and 3.4 for Italian respondents. Given the scale for association strengths is a five point Likert scale, the results confirmed the relevance of these identified associations. It is interesting to note that “A country between mountains and lakes”, and “Beautiful landscape, scenic views” are among the top five association strengths for both markets. On the other hand, “Boring”, and “Festivals and Carnival” were perceived as less associated with Switzerland tourism.

The overall association valences are 1.45 for French residents, and 1.55 for Italian respondents. The association valences were measured with a seven point (−3 to 3) Likert scale. The positive association valence indicates the overall positive impressions with Switzerland as a travel destination. The attributes of “Beautiful landscape, scenic views” and “Fresh air, clear water, and clean environment” are among the top five attribute valence for these markets. Alternatively, “Boring” and “Expensive” received the lowest valence from both markets reflecting the negative attitudes associated with these statements.

**Table 2.** Means of association strengths and association valences and destination imagery diagnosis quadrant

	France			Italy		
Statements	AS	AV	Q	AS	AV	Q
1. A country between mountains and lakes	4.47	2.49	1	4.09	2.20	1
2. Safe	3.76	2.30	1	3.87	2.46	1
3. Multiculturalism, multi-lingual	3.65	1.95	1	3.13	1.76	2
4. Offers many outdoor activities with amazing views	3.84	2.08	1	3.59	2.05	1
5. Enjoy nature, connect with nature	3.79	2.34	1	3.44	1.94	1
6. Fondue, raclette, cheese, and chocolate	4.13	2.00	1	3.75	1.88	1
7. Beautiful landscape, scenic views	4.34	2.77	1	3.91	2.57	1
8. Precision and organized	3.44	1.22	3	4.13	2.09	1
9. Easy and efficient transportations throughout the journey	3.28	2.08	2	3.64	2.42	1
10. Transportation is a travel experience itself	2.88	1.45	3	3.02	1.75	2
11. Festivals and Carnival	2.83	1.30	3	2.15	1.00	3
12. St. Moritz, Zermatt, and Matterhorn	2.72	0.81	3	3.05	0.93	3
13. Calm, peaceful, tranquil	4.01	2.14	1	3.82	2.02	1
14. Authentic untouched nature	3.83	2.53	1	3.60	2.38	1
15. Fresh air, clear water, and clean environment	4.15	2.61	1	3.75	2.55	1
16. Expensive	4.06	−1.84	4	3.56	−1.35	4
17. Money or bank	3.66	−0.40	4	3.86	−0.03	4
18. Boring	1.72	−1.90	3	1.64	−1.97	3
19. Rule based country, strict	3.28	−0.01	3	3.40	0.77	3
20. Switzerland is picturesque	3.71	2.01	1	3.06	1.67	2
21. Healthy life style	3.20	1.65	2	3.21	1.80	2
22. Welcoming or friendly	3.14	2.16	2	2.83	2.06	2
23. Quiet and discreet	3.70	1.71	1	3.68	1.92	1
Average	3.55	1.45		3.4	1.55	

### Destination Imagery Diagnosis Model

To understand the marketing opportunities, the overall averages of the association valence and the association strength were used as the intersection point of the x axis and y axis, respectively, and four quadrants were created, which is the Destination Imagery Diagnosis Model. The Destination Imagery Diagnosis consists of four quadrants (clockwise):

- First Quadrant: Strong association strength, and positive valence. Hence, it is named Treasures.
- Second Quadrant: Weak association strength, but positive valence. Its name is Hidden Gems.
- Third Quadrant: Weak association strength, and negative valence. Consequently, the name is Traps.
- Fourth Quadrant: Strong association strength, but negative valence. It is named as Roadblocks.

**Fig. 1.**
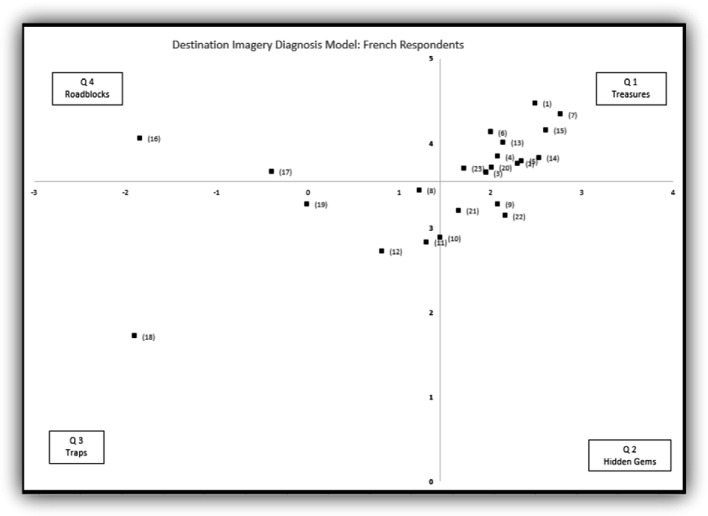
Destination Imagery Diagnosis model for the French market. X-axis: association valence, Y-axis: association strength. Please refer to Table 2 for specific items.

**Fig. 2.**
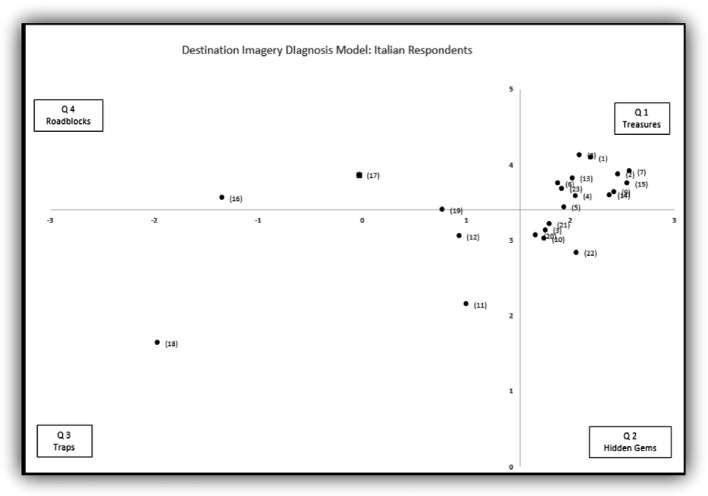
Destination Imagery Diagnosis model for the Italy market. X-axis: association valence. Y-axis: association strength. Please refer to Table 2 for specific items.

Three Destination Diagnosis Models were created and presented in Fig. 1 for French respondents, and Fig. 2 for Italian respondents.

First Quadrant, Treasures: Associations located in the first quadrant are strong and positive. A total of ten associations are located in the first quadrant for these markets. These ten statements are “A country between mountain and lakes”, “Safe”, “Offers many outdoor activities”, “Enjoy nature, connect with nature”, “Fondue, raclette, cheese, and chocolate”, “Beautiful landscape, scenic views”, “Clam, peaceful, tranquil”, “Authentic untouched nature”, “Fresh air, clear water, and clean environment”, and “Quiet and discreet”. It is interesting to note that the findings here echo with the official Switzerland Tourism slogan “Switzerland, get nature”. Many of these strong and positive associations are related to Switzerland’s nature, including mountains, lakes, and implied opportunities for outdoor activities. Marketers should leverage these treasures in their marketing communication.

Second Quadrant, Hidden Gems: Two associations, “Healthy life style” and “Welcoming and friendly” are located here for these markets. Associations located in this quadrant are positive but weak. Hence, marketers should strive to build a stronger association in these markets.

Third Quadrant, Traps: Associations located in this quadrant are weak and less positive or negative. Four associations including “Festivals and carnival”, “St. Moritz, Zermatt, and Matterhorn”, “Boring”, and “Rules based country, strict” are in this quadrant. It is important to point out that the association valances for these association could be positive, but less than associations located in the first and second quadrants. For example, “Festivals and carnival” and “St. Moritz, Zermatt, and Matterhorn” were perceived as positive, while “Boring” was negative, but “Rules based country, strict” was ranked negative by French respondents yet positive by Italian respondents. Association located in this quadrant are less valuable to marketers.

Fourth Quadrant, Roadblocks: Associations located in this quadrant are strong but less positive or negative. In this study, “Expensive” and “Money or Bank” are located in this quadrant for both markets. Switzerland is known for its high living costs. To respond to this strong and negative association, marketers could actively propose more affordable travel alternatives, such as public transportation passes, or alternative accommodations. Overall, marketers should address associations located in this quadrant cautiously as their association valences are not all positive.

The above findings applied to both markets. Furthermore, marketers can use the findings to develop individual marketing strategies for each market. The French market considers “Multiculturalism, multi-lingual”, and “Switzerland is picturesque” as strong and positive, while “Easy and efficient transportation” as positive but weak. Whereas the Italian market “Precision and organized”, and “Easy and efficient transportations” are located in the first quadrant; and “Multiculturalism, multi-lingual”, “Transportation is a travel experience itself”, and “Switzerland is picturesque” are located in the second quadrant. Although similar statements were located in either the first or second quadrants, these markets have different association strengths and valences, and demand different marketing promotion strategies.

## Conclusions/Implications

This research investigated destination imagery of Switzerland as a travel destination, and proposed the Destination Imagery Diagnosis model to analyse the association strengths and association valences and develop corresponding marketing strategies. Associations located in the third and fourth quadrants are similar in the sense that they are less positive or negative, but require different marketing strategies. Associations located in the fourth quadrant are strongly associated with the destination, hence marketers could either actively correct these less positive associations, or passively leave these associations along. On the other hand, associations located in the third quadrants are weakly associated with the destination, and probably should not be the top priority for marketers.

For academia, this research applied the Destination Imagery definition proposed by [6, 8], conducted empirical research to investigate the destination imagery in two target markets, and analysed the association strengths and valences. This research contributes to the academia by proposing the Destination Imagery Diagnosis Model, which could be used to analyse destination imagery, compare perceptions between markets as shown in this research, and develop marketing strategies. For practitioners, this research contributes by illustrating the Destination Imagery Diagnosis model with data collected from France and Italy, and provides examples of marketing insights for destination marketing organizations.

This research is not without limitation. This research respondents were recruited from Prolific, an online platform for online subject recruitment focusing on academic research. The reliability of Prolific has been examined and documented [12]. Nevertheless, the age groups of this research respondents are mainly between 18 and 39 years old, and may not correspond to the French and Italian tourist profiles for Swiss Tourism. Hence, this could be considered as a research limitation. This research collected demographic data including age, gender, and previous travel experience, but is in the process of analysing the data. Hence, the potential impact of age, gender, or travel experience could not be reported here. The data collection of this research took place in January 2020 before the COVID-19 Pandemic. Future research could refer to this research and compare the Destination Imagery Diagnosis Model for Switzerland after the Pandemic. Future research is also encouraged to apply the Destination Imagery Diagnosis model to other destinations, especially competing destinations sharing similar stereotypes or tourism attractions. Additionally, this research did not focus on the projected destination image, nor incorporated the induced information sources. Future researchers are encouraged to compare the Destination Imagery Diagnosis models developed based on different information sources [11], and between projected and perceived destination images.
